# Deciphering the heterogeneity of neutrophil cells within circulation and the lung cancer microenvironment pre- and post-operation

**DOI:** 10.1007/s10565-024-09850-z

**Published:** 2024-02-06

**Authors:** Fangming Liu, Xuanqi Liu, Yifei Liu, Dongsheng Chen, Xiaoxia Liu, Chuan Qin, Yuanlin Song, Hao Fang, Duojiao Wu

**Affiliations:** 1 Center for Tumor Diagnosis and Therapy, Jinshan Hospital, Fudan University, Shanghai, China; 2 Shanghai Institute of Clinical Bioinformatics, Shanghai, China; 3 Institute of Clinical Science, Zhongshan Hospital, Fudan University, Shanghai, China; 4 Center of Molecular Diagnosis and Therapy, The Second Attached Hospital of Fujian Medical University, Quanzhou, China; 5 Suzhou Institute of Systems Medicine, Suzhou, Jiangsu Province China; 6 Respiratory Department, Zhongshan Hospital, Fudan University, Shanghai, China; 7 Department of Medical Ultrasound, Jinshan Hospital, Fudan University, Shanghai, China; 8 Department of Anesthesiology, Shanghai Geriatic Medical Center, Shanghai, China; 9 Department of Anesthesiology, Zhongshan Hospital, Fudan University, Shanghai, China

**Keywords:** Neutrophils, Single-cell sequencing, NSCLC, Heterogeneity

## Abstract

Neutrophils play a crucial role in the immune system within tumor microenvironment. At present, numerous studies have explored the changes of neutrophils’ automatic killing effect and cellular communication with other immune cells under pathological conditions through single-cell sequencing. However, there remains a lack of definite conclusion about the identification criteria of neutrophil subgroups. Here, we collected tumor and para-carcinoma tissues, pre- and postoperative blood from patients with non-small cell lung cancer (NSCLC), and performed single-cell RNA (scRNA) sequencing to evaluate the distribution of neutrophil subgroups. We have developed a computational method of over expression rate (OER) to evaluate the specificity of neutrophil subgroups, in order to target gene panels with potential clinical application value. In addition, OER was used to evaluate specificity of neutrophil subsets in healthy people and patients with various diseases to further validate the feasibility of this evaluation system. As a result, we found the specificity of Neu_ c1_ IL1B and Neu_ c2_ cxcr4 (low) in postoperative blood has increased, while that of IL-7R + neutrophils has decreased, indicating that these groups of cells possibly differentiated or migrated to other subgroups in the state of lung cancer. In addition, seven gene panels (Neu_c3_CST7, RSAD2_Neu, S100A2/Pabpc1_Neu, ISG15/Ifit3_Neu, CD74_Neu, PTGS2/Actg1_Neu, SPP1_Neu) were high specific in all the four NSCLC-associated samples, meaning that changes in the percentage of these cell populations would have a high degree of confidence in assessing changes of disease status. In conclusion, combined consideration of the distribution characteristics of neutrophil subgroups could help evaluate the diagnosis and prognosis of NSCLC.

**Graphical abstract:**

• An evaluation system based on OER was developed to assess the specificity of neutrophil subgroups

• Specificity of Neu_ c1_ IL1B, Neu_ c2_ cxcr4 (low) and IL-7R + neutrophils changed significantly between preoperative and postoperative blood

• 7 gene panels were high specific in all the four NSCLC-associated samples, meaning a high degree of confidence in assessing changes of these subgroups in various disease status

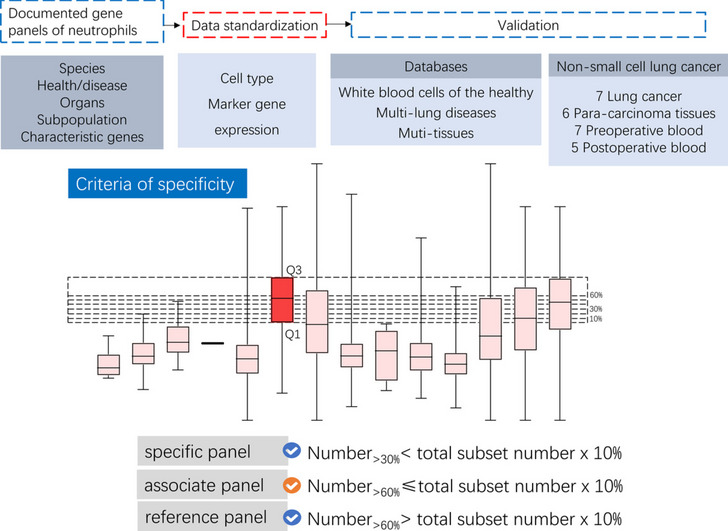

**Supplementary Information:**

The online version contains supplementary material available at 10.1007/s10565-024-09850-z.

## Introduction

Neutrophils are vital immune cells in the human body that play a role in fighting various pathogens, including bacteria, fungi, and protozoa. Neutrophils account for 50–70% of all circulating leukocytes (Mayadas et al. 2014), which constitute the first line of defense of the body. After being recruited to the infection site, neutrophils recognize and interact with pathogenic microorganisms through three major methods, which are phagocytosis, degranulation, and the formation of neutrophil extracellular traps (NETs) (Alasmari 2020; Chen et al. 2018). In addition to their autologous killing effect, there are also important interactions between neutrophils and other immune cells. Neutrophils communicate with macrophages, dendritic cells (DCs), and adaptive immune cells through direct cell–cell contact or soluble mediators. For example, NETs derive proinflammatory through release of cathepsin B from lysosomes, formation of pyroptosome, caspase-1 activation, and pyroptosis of macrophages (Chen et al. 2018). A group of neutrophils are defined as B-cell-helper neutrophils (NBH) in the area around the follicles in the spleen of humans, mice, and rhesus monkeys, which can directly regulate the biological process of B cells and promote their survival and differentiation by producing vital cytokines such as B-cell activating factor (BAFF) and a promotion inducing light (APRIL) (Costa et al. 2019). A recent study (Chen et al. 2021) demonstrated that free fatty acids (FFAs) could induce the release of NETs by increasing the expression of ERK, p38, and JNK. FFAS-induced NETs could promote the activation of DCs, thereby inducing the differentiation of primary CD4^+^ T cells into Th1 and Th17 cells, which followed by release of IL-1β, IL-12, and TNF-α.

Single-cell RNA sequencing (scRNA-seq) has experienced a development of a blowout type during the last years. scRNA-seq has considerable advantages in exploring the heterogeneity of tumor cells or immune cells (Kinker et al. 2020; Papalexi and Satija 2018; Patel et al. 2014). In the process of tumor genesis and development, tumor cells obtained different genetic or epigenetic characteristics, which lead to differences in tumor proliferation, migration and drug sensitivity within and between tumors (Fan et al. 2019; Lim and Ma 2019; Lüönd et al. 2021), and variable responses to traditional therapy and immunotherapy (Lee et al. 2018; Wu et al. 2021). In addition, some rare cell populations may also be the potential factors for tumor metastasis and drug resistance (Grün et al. 2015; Lim and Ma 2019). The characterization of tumor heterogeneity will facilitate effective targeted therapy and find new ways to overcome the immunosuppression of tumor infiltrating immune cells (Chung et al. 2017).

In consideration of the key roles of neutrophils in innate immunity and adaptive immunity, studies of tumor infiltrating neutrophils at single-cell resolution have been operated. For example, recent evidence (Wang et al. 2022) suggested that in the tumor microenvironment of patients with pancreatic ductal adenocarcinoma, glycolysis signature was upregulated along differentiation process of neutrophils, and was hyperactivated in terminally differentiated pro-tumor subpopulation (TAN-1), which was associated with poor prognosis. It has been proved by further research that BHLHE40 was the key regulatory factor that mediate polarization of neutrophils towards TAN-1. As for lung cancer, a subgroup of neutrophils was defined as tissue-resident neutrophils (TRNs) through scRNA-seq (Salcher et al. 2022). A series of genes that are highly specific for TRNs (also known as gene signature) were finally considered to be associated with anti-PD-L1 therapy failure. Similarly, it was suggested that tumor-associated neutrophils (TANs) of liver cancer patients inhibited cytotoxic CD8^+^ T cell activity through high expression of PD-L1 (Xue et al. 2022). In addition, CCL4^+^TANs highly expressed chemokines CCL3 and CCL4, which had a recruitment effect on macrophages. This study revealed the heterogeneity of neutrophils in liver cancer, indicating the cancer-promoting role of most tumor-associated neutrophils. Although increasing attention has been paid to the immune function of neutrophils in the tumor microenvironment, changes in the subtype of tumor infiltrating neutrophils are still poorly understood (Salcher et al. 2022). The identification of heterogeneity of neutrophil subpopulations is a prerequisite for exploration of their immune functions.

The aim of this manuscript is to evaluate the heterogeneity of neutrophils in tumor tissue, para-carcinoma tissue, and preoperative and postoperative blood in patients with non-small cell lung cancer (NSCLC). Common or specific neutrophil subsets were identified in different organs or at different time points, as well as specific changes of the same subsets in different environments. We have developed a methodology to measure specificity of subpopulations, in order to target neutrophil subsets that are clinically valuable for treatment and prognosis.

## Methods

### Human specimens

Tumor and adjacent normal lung tissues and peripheral blood of patients who were pathologically diagnosed with NSCLC were obtained during 2020. Tumor tissues were obtained from seven patients and normal lung tissues were collected from six patients through surgical removement. Peripheral blood was collected before surgical procedure, namely preoperative blood, and 3 days after surgery, defined as postoperative blood. Seven preoperative blood and five postoperative blood samples were included in this research. A total of 14 patients including four females and ten males aged between 47 and 78 years participated in the study; they were pathologically diagnosed with squamous cell carcinoma (*n* = 3), adenocarcinoma (*n* = 10), and large cell carcinoma (*n* = 1). This study was approved by the Ethics Committee of Zhongshan Hospital, Fudan University. All patients in this study provided written informed consent for sample collection and data analyses.

### Preparation of single-cell resuspension of tissues

Fresh tissues were immersed in phosphate-buffered solution (PBS) containing 1% fetal bovine serum (FBS). The tissues were cut into 1–2 mm tissue blocks with surgical scissors, which were then put into a tissue grinder to grind into a paste. 1%FBS-PBS was added again to resuspend the tissue sufficiently, then the single cell suspension was obtained through a 70-um filter. After centrifugation at 1100 rpm for 10 min, the cell precipitates were resuspended with RBC lysate, following with incubation at room temperature for 5 min and cleaning with PBS solution.

### Preparation of white blood cells (WBC)

The whole blood was centrifuged at 3000 rpm for 10 min, followed by removement of the supernatant. The rest part was mixed with ACK Lysis Buffer at a ratio of 1:3, then gently swirl or mix the tube upside down. Leave it at room temperature for 5 min, gently swirling and mixing twice. The supernatant was removed by centrifugation and the above lysis steps were repeated two to three times until the red blood cells in the cell precipitation were completely removed. Centrifuge at 4 ℃, 450 g for 10 min to precipitate white blood cells, carefully absorb and discard the supernatant. Resuspended cells were used for subsequent single cell sequencing.

### Single-cell RNA sequencing

Single cell suspensions of tissues and white blood cells were soaked in stain buffer for the next step. First of all, cells were marked with DRAQ7 and Calcein AM, which are used for cell activity detection. The quality of cell suspensions was judged by scanning imaging. Cells were ready for the next step with the activity greater than 80% and no interference from cell debris, platelets, and red blood cells. If the activity was less than 80%, the living cells were sorted by Dead Cell Removal Kit. Samples that have subjected to quality control were marked with sample tags. In brief, cells that more than 5 × 10^5 and no more than 1 × 10^6 was prepared and resuspended in 180-µl stain buffer, mixing with 20-µl sample tag, and incubated for 30 min on ice. Then the mixture was washed twice using stain buffer and resuspended in sample buffer. Quality control step was performed again to reconfirm cell viability and cell concentration. The process of obtaining single cell nucleic acid was carried out on the BD Rhapsody platform using BD Rhapsody Cartridge Kit and cDNA kit. Briefly, different samples were mixed according to the volume calculated by scanner. Then the cell load step was carried out, which was to randomly distribute a single cell into the microwell in the chip (about 200,000 microwells). After incubated at room temperature for 15 min, cells that have not settled in the microwells were removed, and then the capture beads were loaded into the chip. Each microwell can hold one magnetic bead and one cell. Capture beads contains barcode and UMI sequences that can help identify different cells and different transcripts. After the capture beads were fully settled in the chip, the free magnetic beads were washed out. Cells in the chip were lysed and then the magnetic beads combined with transcripts were quickly collected. The obtained magnetic beads were reverse transcribed within 30 min, in which each cDNA molecule was labeled with UMI and barcode so that its source cells can be recognized. The mRNA Whole Transcriptome Analysis (WTA) and Sample Tag Library Preparation were then performed using BD Rhapsody WTA Amplification Kit. In brief, the capture beads were subject to random priming and extension, WTA Index PCR, Sample Tag PCR, and Sample Tag Index PCR. The obtained Sample Tag libraries and WTA libraries were subject to quality control by Agilent 2100 Bioanalyzer. Sequencing was done through the Illumina NovaSeq6000 platform, reading length as double-ended 150 bp.

### Data information

We also collected scRNA-seq data from the databases for healthy people, patients with lung diseases, and other types of diseases. ScRNA-seq data of peripheral leukocytes of the healthy was collected from GSM5676985, GSM5676986, GSM5676987, GSM5676988, GSM5676989, GSM5676990, detailed information was included in Table S1.1. Lung disease datasets were obtained from GSE136831, GSE128169, GSE128033, GSE131907_Lung_Cancer, E-MTAB-6653 and E-MTAB-6149 (Table S1.2). We extracted sequencing information of 146 subjects from these six datasets, including 49 healthy controls (NOR), seven para-carcinoma tissues (PC_NOR), 18 chronic obstructive pulmonary disease (COPD) patients, 40 idiopathic pulmonary fibrosis (IPF) patients, eight systemic sclerosis (SSC) patients, and 24 lung adenocarcinoma (LUAD) patients. In addition, 36 diseases of various organs have also been downloaded from public databases; detailed information was shown in Table S1.3.

### Statistical analysis

The gene expression matrix generated by sequencing was analyzed by R4.1.1 and Rstudio software. Firstly, Seurat R toolkit was used to conduct data quality control. Cells with detected genes less than 500 or more than 7000 were excluded, and cells with mitochondrial gene expression more than 20% were filtered out. The filtered data was normalized and dimensionally reduced by FindVariableFeatures, NormalizeData, ScaleData and RunPCA functions. The samples were further integrated by findintegrationanchor and IntegrateData functions. The gene expression of clusters was referred to further determine the cell categories.

Previously, an evaluation procedure developed by our group was used to further evaluate the specificity of cell clusters (Liu et al. 2022). In this paper, we collected 78 gene sets that defined different neutrophil populations from published data (Table S2), counted the number of positive cells for every cluster, differential expression genes, and Standard Error of Mean of gene expression. A series of cut off values were set to calculate the over expression rate (OER) of a gene set, which was used to assess the specificity of this gene set in other neutrophil subsets. For example, the highest expression of a gene set in its own subpopulation was recorded as Q3, while the lowest expression was Q1. Cut off (10%) was obtained by (Q3-Q1)/10 + Q1, cut off (20%) was obtained by (Q3-Q1)/10 × 2 + Q1, and so on. If the expression level of the same gene set in other neutrophil subsets was greater than the cut off value, it was denoted as 1; otherwise, it was denoted as 0. The number of subsets denoted as 1 was recorded as *subset number*. We counted the subset numbers in different cut off intervals, that was, less than 10%, 10–20%, 20–30%, 30–40%, 40–50%, 50–60%, and more than 60%. The number of cell populations assessed in the NSCLC samples was called the *total subset number*, and the percentage of subset number in the total subset number was defined as OER.

Based on the specificity of gene panel that used to define neutrophil subsets, all the subgroups were divided into three classes, which was specific panel, associate panel, and reference panel. The measuring standard was as follows. Specific panel, subset number with cut off value more than 30% < (total subset number × 10%). Associate panel, subset number with cut off value more than 60% ≤ (total subset number × 10%). Reference panel, subset number with cut off value more than 60% > (total subset number × 10%), except for specific panels.

## Results

### Neutrophil subsets in healthy peripheral blood

**Fig. 1:**
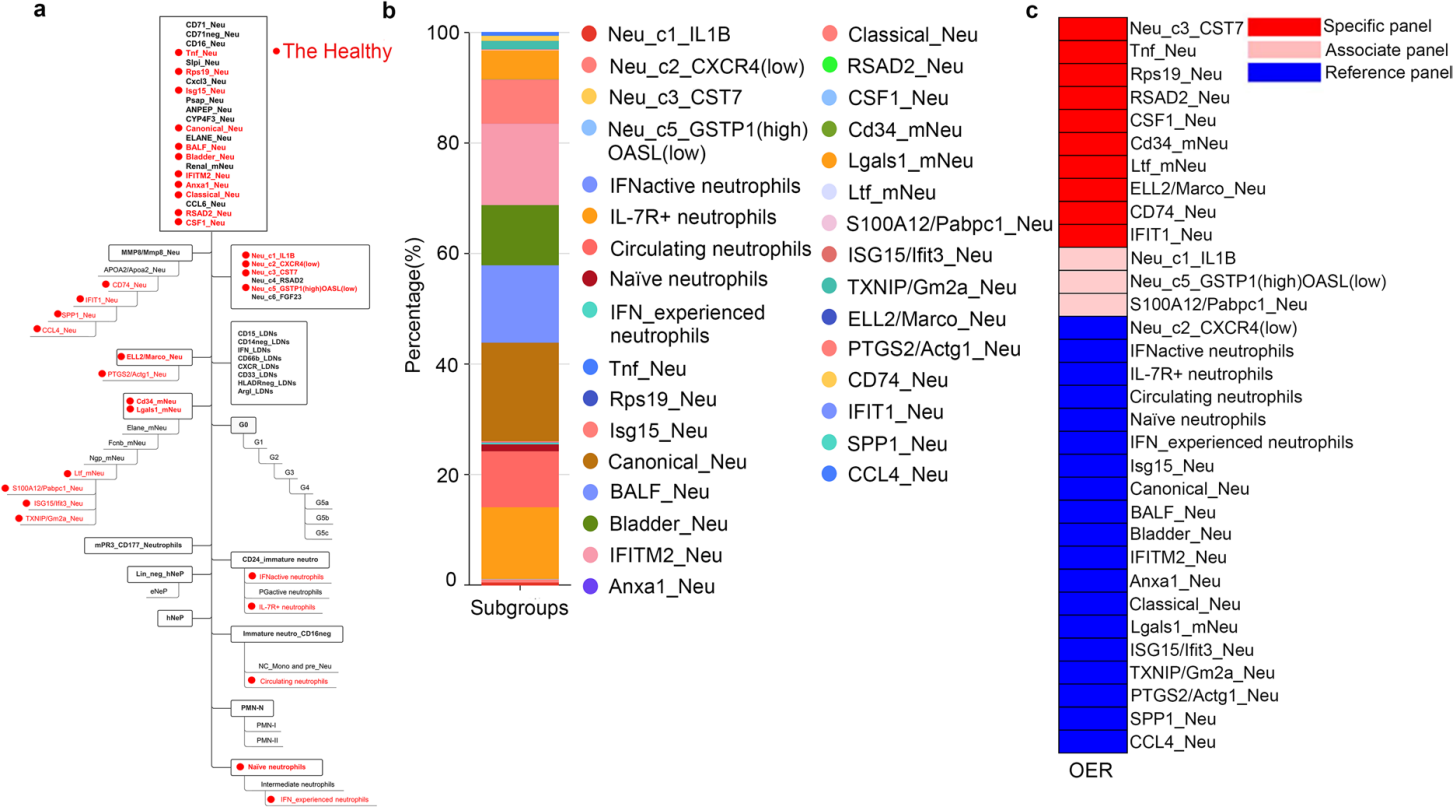
Distribution of neutrophil subsets in peripheral blood of six healthy donators. **a** Neutrophil subpopulations in hierarchy chart. Detected subgroups was marked in red. **b** Proportion of neutrophil subgroups of the healthy. **c** Specificity of detected neutrophil subsets

We collected 78 gene panels published in literatures for the identification of neutrophils (Table S2). Among these panels, some of them were used to assess neutrophils in body fluids, such as peripheral blood, bone marrow, and bronchoalveolar lavage fluid. The other gene panels were used to evaluate neutrophil subsets in the tissue. Some of these gene panels were obtained from healthy or control samples, while some were from disease samples. Part of these gene sets were universal, meaning they can be used to evaluate both healthy samples and disease samples, or exist in both body fluid samples and tissue samples. The collected cell subsets were divided into several different hierarchies, some of which formed differentiation tracks among the cell subsets (Fig. 1a).

scRNA-seq data of peripheral blood cells from six healthy donors was obtained from published datasets. There were 32 neutrophil populations in all of the specimens, which were marked in red in Fig. 1a. Among them, Canonical_Neu occupied the largest proportion (Table S3), which was characterized by high expression of CSF3R, S100A8 and S100A9, indicating that this cell subpopulation performs essential functions of neutrophils, such as differentiation, cell adhesion, or recognition processes. In terms of proportion, it was closely followed by IL-7R + neutrophils, BALF_Neu and IFITM2_Neu. The specificity of 32 gene panels was evaluated according to OER referred above (Fig. 1c). As a result, ten specific panels and three associate panels were recognized (Tables 1, 2 and 3 and Table S4), illustrating that these 13 gene panels were relatively high reliable as clustering criteria in the healthy.

**Table 1:** OER of cell-specific panel in peripheral blood of the healthy

	OER_<10_	OER _10–20_	OER_20-30_	OER_30-40_	OER_40-50_	OER_50-60_	OER_>30_	OER_>60_
Neu_c3_CST7	96.88	0	0	0	0	0	3.12	3.12
Tnf_Neu	96.88	0	0	0	0	0	3.12	3.12
Rps19_Neu	96.88	0	0	0	0	0	3.12	3.12
RSAD2_Neu	96.88	0	0	0	0	0	3.12	3.12
CSF1_Neu	96.88	0	0	0	0	0	3.12	3.12
Cd34_mNeu	96.88	0	0	0	0	0	3.12	3.12
Ltf_mNeu	96.88	0	0	0	0	0	3.12	3.12
ELL2/Marco_Neu	90.62	3.12	0	0	0	0	6.25	6.25
CD74_Neu	96.88	0	0	0	0	0	3.12	3.12
IFIT1_Neu	96.88	0	0	0	0	0	3.12	3.12

**Table 2:** OER of cell-associate panel in peripheral blood of the healthy

	OER_<10_	OER _10–20_	OER_20-30_	OER_30-40_	OER_40-50_	OER_50-60_	OER_>30_	OER_>60_
Neu_c1_IL1B	40.62	15.62	21.88	9.38	3.12	3.12	21.88	6.25
Neu_c5_GSTP1(high)OASL(low)	81.25	0	0	3.12	3.12	3.12	18.75	9.38
S100A12/Pabpc1_Neu	90.62	0	0	0	0	0	9.38	9.38

**Table 3:** OER of cell-reference panel in peripheral blood of the healthy

	OER_<10_	OER _10–20_	OER_20-30_	OER_30-40_	OER_40-50_	OER_50-60_	OER_>30_	OER_>60_
Neu_c2_CXCR4(low)	25	18.75	3.12	0	3.12	21.88	53.12	28.12
IFNactive neutrophils	81.25	0	0	0	0	0	15.62	15.62
IL-7R + neutrophils	84.38	0	0	0	3.12	0	15.62	12.5
Circulating neutrophils	62.5	0	0	0	0	12.5	37.5	25
Naïve neutrophils	18.75	0	0	3.12	6.25	34.38	81.25	37.5
IFN_experienced neutrophils	84.38	0	0	0	0	0	15.62	15.62
Isg15_Neu	84.38	0	0	0	0	0	15.62	15.62
Canonical_Neu	37.5	3.12	0	6.25	0	6.25	59.38	46.88
BALF_Neu	50	0	3.12	3.12	3.12	0	46.88	40.62
Bladder_Neu	56.25	3.12	3.12	0	0	6.25	37.5	31.25
IFITM2_Neu	50	0	0	3.12	3.12	0	50	43.75
Anxa1_Neu	71.88	0	3.12	0	3.12	0	25	21.88
Classical_Neu	37.5	3.12	3.12	15.62	15.62	6.25	56.25	18.75
Lgals1_mNeu	50	6.25	0	3.12	0	3.12	43.75	37.5
ISG15/Ifit3_Neu	84.38	0	0	0	0	0	15.62	15.62
TXNIP/Gm2a_Neu	37.5	9.38	6.25	12.5	9.38	9.38	46.88	15.62
PTGS2/Actg1_Neu	75	6.25	3.12	0	3.12	0	15.62	12.5
SPP1_Neu	75	0	0	0	0	0	21.88	21.88
CCL4_Neu	81.25	0	0	3.12	0	0	18.75	15.62

### Neutrophil subsets in NSCLC

**Fig. 2:**
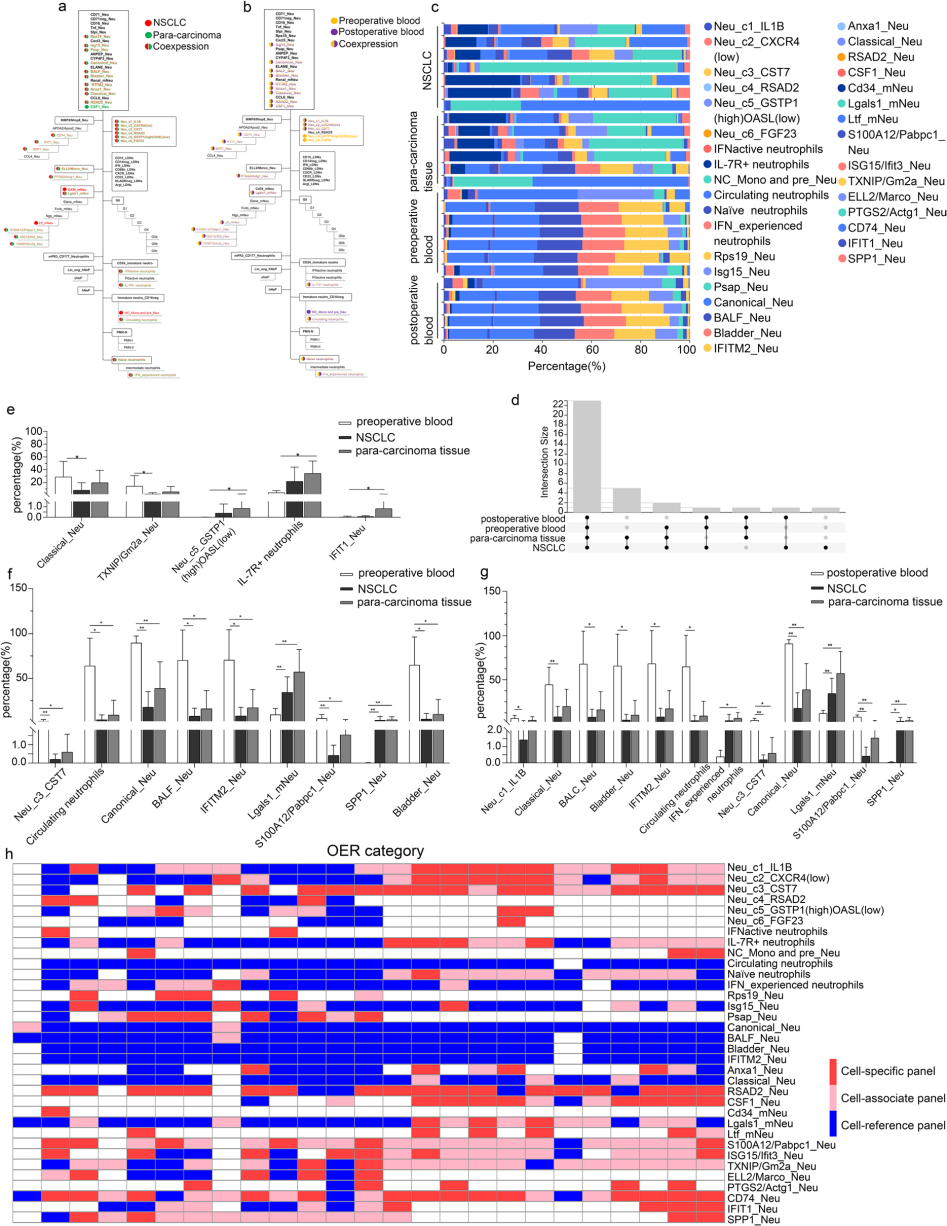
Proportion and specificity of neutrophil subsets in NSCLC. **a** Detected neutrophil subpopulations of NSCLC and para-carcinoma tissue in hierarchy chart, which were marked in red and green, respectively. **b** Detected neutrophil subpopulations of preoperative and post operative blood in hierarchy chart, which were marked in yellow and purple, respectively. **c** Proportion of neutrophil subgroups. **d** Co-expressed subgroups in NSCLC. The number of shared cell groups was displayed on the Y axis. **e**–**g** Neutrophil subpopulations with significant differences in the expression of NSCLC, para-carcinoma tissue, preoperative and postoperative blood. **h** Specificity of neutrophil subgroups in NSCLC. Specific panel, associate panel and reference panel were exhibited as blue, pink, and red, respectively

We evaluated the distribution of neutrophils by scRNA-seq in seven tumor tissues, six adjacent normal lung tissues, seven preoperative blood, and five postoperative blood samples from patients with NSCLC. Based on the previously established evaluation procedure, we found that several types of neutrophils were prevalent in the tissues and blood of lung cancer patients, namely Neu_c1_IL1B, Neu_c2_cxcr4(low), Neu_c3_CST7, IL-7R + neutrophils, Circulating neutrophils, Naïve neutrophils, IFN_experienced neutrophils, mNeu_14_ Lgals1, S100A12/Pabpc1_Neu, TXNIP/Gm2a_Neu, CD74_Neu and several general neutrophil groups (Fig. 2a-c, Table S5), whereas CD34_mNeu was expressed specifically in NSCLC. Figure 2d exhibited statistical overview of co-expressed subpopulations of neutrophils. Among these populations, there were significant differences in expression of Classical_Neu and TXNIP/Gm2a_Neu between preoperative blood and NSCLC, while the proportion of Neu_c5_GSTP1(high)OASL(low), IL-7R + neutrophils, and IFIT1_Neu was significantly different between preoperative blood and para-carcinoma tissue (Fig. 2e). In addition, there were nine subpopulations whose proportion was significantly different between preoperative blood and both the tissues (Fig. 2f). The distribution of several subsets was also comparable between postoperative blood and tissues, showed in Fig. 2g.

The distribution specificity of neutrophil subsets was assessed according to the criteria mentioned above (Fig. 2h, Table S6-S9). Although Neu_c1_IL1B and Neu_c2_cxcr4(low) are widely exist in lung cancer, para-carcinoma tissue, preoperative and postoperative blood from NSCLC patients, the specificity of their distribution varied significantly. In peripheral blood, especially postoperative blood, the increased specificity of these two populations suggests that they are more credible as prognostic biomarkers of circulatory system (OER_10_ = 96.3%). Proportion of IL-7R + neutrophils in peripheral blood also may be a useful guideline (OER_10_ = 96.43% in preoperative blood, OER_10_ = 88.89% in postoperative blood). It is worth noting that some gene panels were specific or associated in all the four NSCLC-associated samples (Neu_c3_CST7, RSAD2_Neu, S100A2/Pabpc1_Neu, ISG15/Ifit3_Neu, CD74_Neu, PTGS2/Actg1_Neu, SPP1_Neu), meaning that changes in the distribution of these cell populations would have a high degree of confidence in assessing disease changes. The specificity changes between cancer and para-carcinoma tissues or between preoperative and postoperative blood may be due to differentiation or migration of cell subsets in response to changes in the disease microenvironment.

### Neutrophil subsets in lung diseases

**Fig. 3:**
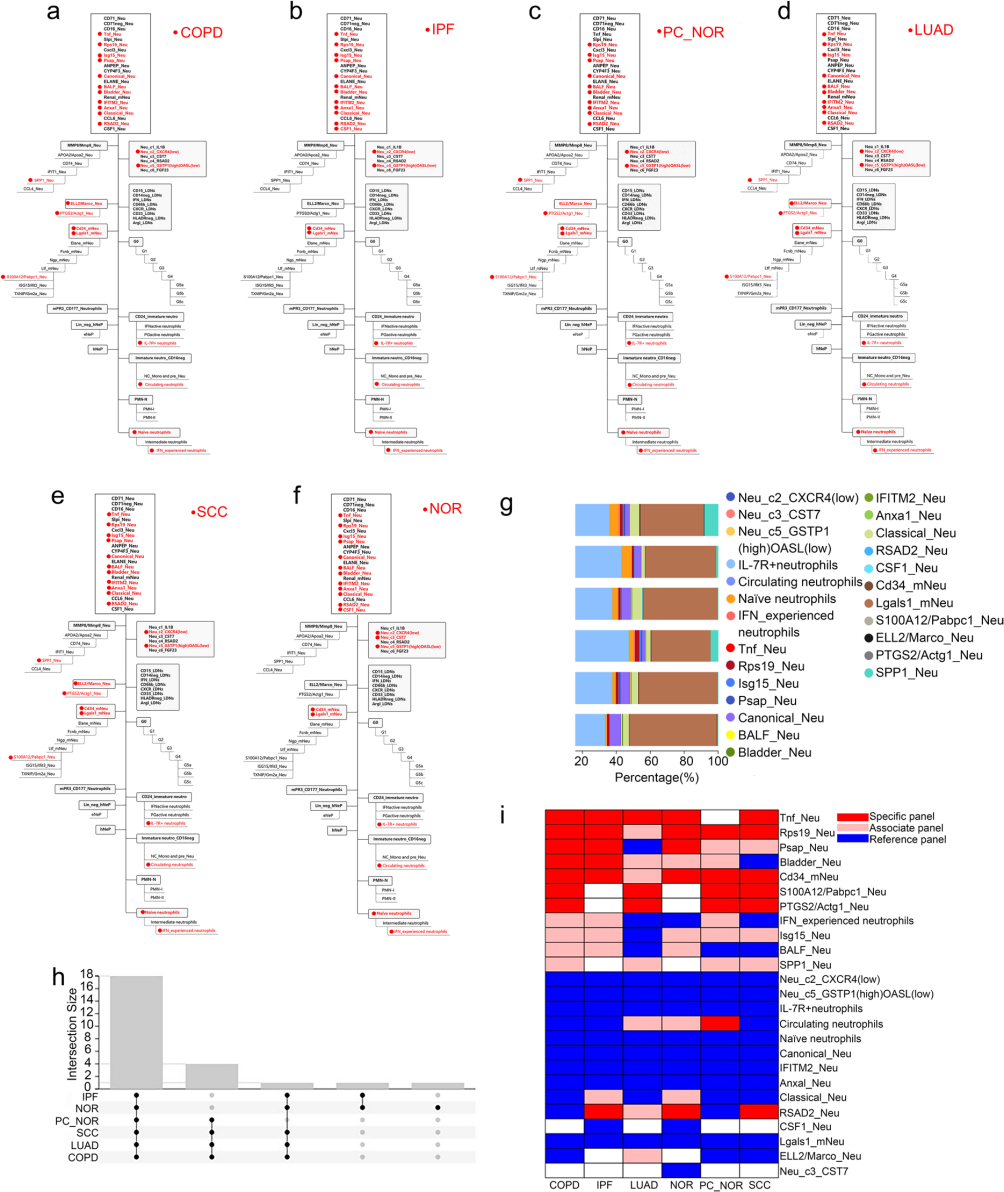
Proportion and specificity of neutrophil subsets in lung diseases, including 49 NOR, 7 PC_NOR, 18 COPD patients, 40 IPF patients, eight SSC patients, and 24 LUAD patients. **a**–**f** Detected neutrophil subpopulations of lung diseases in hierarchy chart. **g** Proportion of neutrophil subgroups. **h** Co-expressed subgroups among different lung diseases. The number of shared cell groups was displayed on the Y axis. **i** Specificity of neutrophil subgroups among lung diseases. Specific panel, associate panel, and reference panel were exhibited as blue, pink, and red, respectively

Then, we obtained the single cell sequencing results of different lung diseases from the GEO database, and also evaluated distribution of the neutrophil populations (Fig. 3a-g, Table S10). A total of 18 subgroups were prevalent in all lung tissue samples. S100A12/Pabpc1_Neu, ELL2/Marco_Neu, SPP1_Neu and PTGS2/Actg1_Neu existed in PC_NOR, COPD, SCC and LUAD. Tnf_Neu was the common cell population of IPF, NOR, SCC, LUAD, and COPD. CSF1_Neu was expressed in both IPF and NOR. NOR specifically expressed Neu_c3_CST7 (Fig. 3h). All of the above information provides a reference for the diagnosis of subtypes of different lung diseases.

Several groups of cells were nonspecifically expressed in each sample, such as Neu_c2_CXCR4(low), Neu_c5_GSTP1(high)OASL(low), IL-7R + neutrophils, Naïve neutrophils, Canonical_Neu, IFITM2_Neu,Anxal_Neu, Lgals1_mNeu. In the contrast, Tnf_Neu, Rps19_Neu and Cd34_mNeu showed high specific expression in various lung diseases and normal tissues (Fig. 3i, Table S11-S16). However, although some cell populations are also commonly distributed in various lung disease samples, their specificities are significantly different. For example, Circulating neutrophils was significantly more specific in LUAD, NOR, and PC_NOR, especially in PC_NOR (OER_10_ = 95.65%). Isg15_Neu and Psap_Neu were decided as reference panels in LUAD, but specificity has been increased in other samples, especially Psap_Neu was specific panel in COPD, IPF and NOR (OER_10_ = 62.5%, 90.48% and 86.36%, respectively). S100A12/Pabpc1_Neu and PTGS2/Pabpc1_Neu was not detected in IPF and NOR, but specificity was significantly specific in COPD, LUAD, PC_NOR and SCC.

### Distribution of neutrophil subsets in diseases of various organs

**Fig. 4:**
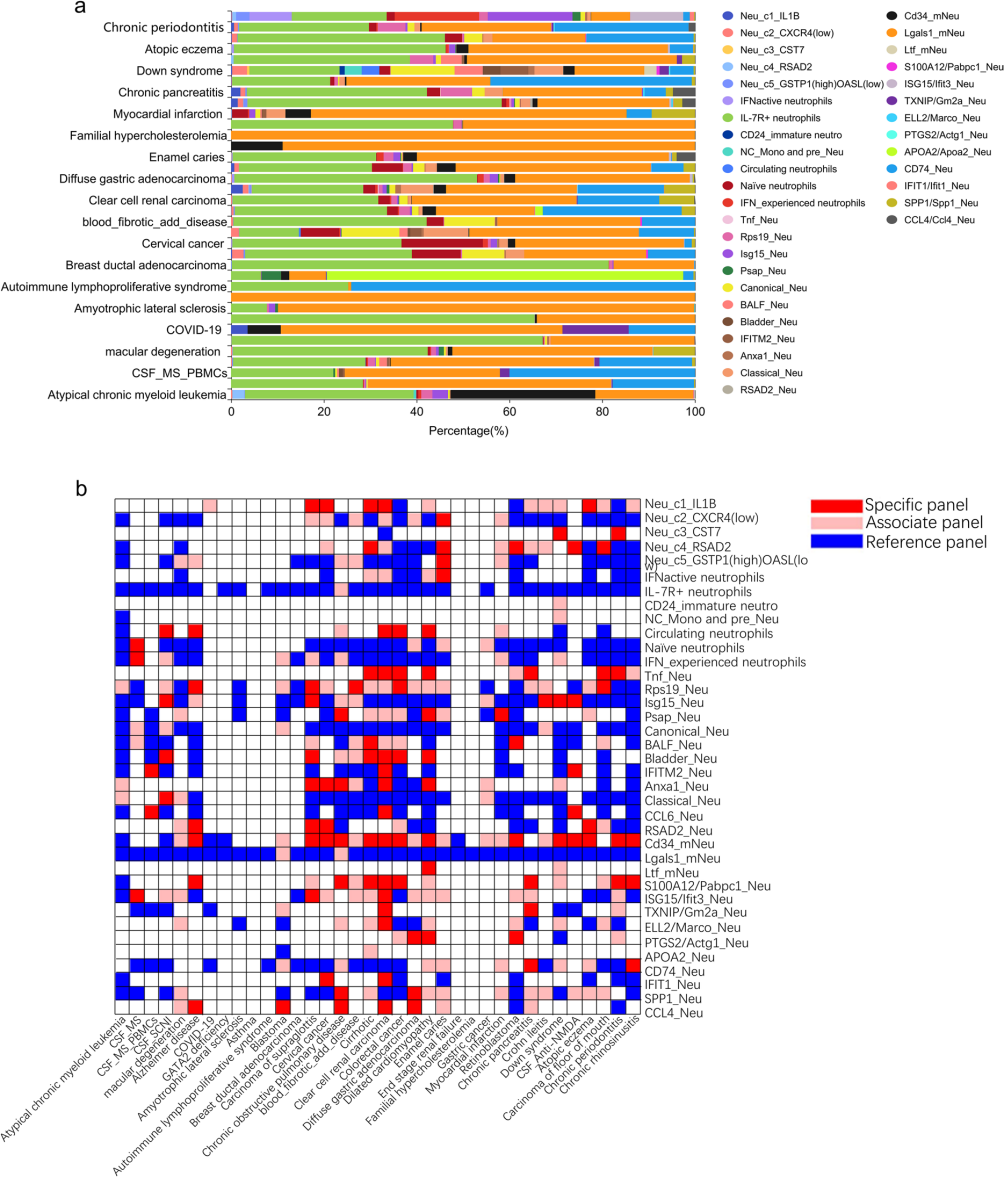
Proportion and specificity of neutrophil subsets in 36 kind of diseases. **a** Proportion of neutrophil subgroups. **b** Specificity of neutrophil subgroups among various diseases. Specific panel, associate panel, and reference panel were exhibited as blue, pink, and red, respectively

The distribution of neutrophil subsets different diseases has been evaluated (Fig. 4a, Table S17). In general, the expression of cell subsets in various organs was extremely different. CD74_Neu, Lgals1_mNeu, and IL-7R + neutrals were the most common subgroups. Among them, IL-7R + neutrophils was a superior cell group in Alzheimer’s disease, GATA2 deficiency with susceptibility to MDSAML, breast ductal adenocarcinoma, diffuse gastric adenocarcinoma, and retinoblastoma. The proportion of Lgals1_mNeu in some diseases was absolutely dominant, such as multiple sclerosis, COVID-19, amyotrophic lateral sclerosis, asthma, enamel caries, end stage renal failure, familial hypercholesterolemia, and myocardial infarction. CD74_Neu was widely present in PBMCs of multiple sclerosis, autoimmune lymphoproliferative syndrome cirrhotic, crohn ileitis, and chronic periodontitis. In addition, there were a large number of Cd34_mNeu in atypical chronic myeloid leukemia. There was a high proportion of IFNactive neutrophils, IFN_experienced neutrophils, Isg15_Neu, and ISG15/Ifit3_Neu in chronic rhinosinusitis. In blastoma, APOA2 _Neu occupied the majority. Comparatively, a larger variety of neutrophil subsets existed in cirrhotic, clear cell renal carcinoma, colorectal cancer, down syndrome, chronic periodontitis, and chronic rhinosinusitis, which indicated a higher heterogeneity of the immune environment.

Then we have compared the distribution of neutrophil subsets from different diseases of the same tissue to evaluate its feasibility in the identification of disease subtypes. Compared with atypical chronic myeloid leukemia and GATA2 deficiency, which are both from bone marrow, atypical chronic myeloid leukemia was also distributed with different proportion of Cd34_mNeu, Rps19_Neu, and Isg15_Neu, in addition to IL-7R + neurophils and Lgals1_mNeu (accounting for almost all the subgroup species of GATA2 defense). Compared with the anti-NMDA receptor encephalitis and the amyotrophic lateral sclerosis, which belong to brain diseases, Lgals1_mNeu accounted for the vast majority of the amyotrophic lateral sclerosis, while the proportion of IL-7R + neutrals in anti-NMDA receiver encephalitis was significantly increased. The distribution of neutrophil subpopulations in diffuse gas adenocarcinoma and gas cancer was similar, as well as crohn ileitis and colonial cancer. Compared with the end stage renal failure and the clear cell renal failure, the neutrophil subgroup of the end stage renal failure was dominated by Lgals1_mNeu, while the main cell subgroups of clear cell renal carcinoma were IL-7R + neutrals, Lgals1_mNeu and CD74_Neu. The distribution of subgroups of familial hypercholesterolemia and blastoma whose specimens were both obtained from liver was also significantly different. The former expressed almost exclusively Lgals1_mNeu, while the main cell group of the latter was APOA2_Neu (Fig. 4a, Table S17).

Finally, we focused on the specificity of the above cell subsets (Fig. 4b. Rps19_Neu, Anxa1_Neu and Classical_Neu had high specificity in the atypical chronic myeloid leukemia, but they were not expressed in GATA2 deficiency. Neu_c4_RSAD2, Isg15_Neu, IFITM2_Neu, CCL6_Neu, and Cd34_mNeu were assessed as specific panels (OER_10_ > 83%, data was not shown) in anti-NMDA receiver encephalitis, which was significantly different from that of amyotrophic lateral sclerosis. Interestingly, the specificity of several subpopulations has changed between diffuse gas adenocarcinoma and gas cancer. Rps19_Neuwas associated panel in the former, and was reference panel in the latter. Anxa1_Neu and Classical_Neu were reference panels in diffuse gas adenocarcinoma, and their specificity in gas cancer was increased to associate panels. The subgroups specificity also changed in the comparison between crohn ileitis and colorectal cancer, where Circulating neutrophils, Psap_Neu, Bladder_Neu, Anxa1_Neu, and SPP1_Neu were more prominent. Last but not the least, the specificity of Cd34_mNeu in clear cell renal cancer was higher than that in end stage renal failure (OER_10_ = 96.77%). These results suggested that the OER-based cell type assessment pattern could be applied to various disease types, not just lung diseases. Because of the specificity of neutrophil distribution in some types of diseases, we speculate that this evaluation system can assist in the identification of disease subtypes to a certain extent.

## Discussion

In this article, we performed single-cell RNA sequencing on cancer tissue, para-carcinoma tissue, preoperative blood, and postoperative blood from patients with non-small cell lung cancer, and investigated the distribution and specific expression of neutrophil subsets using a set of computational methods to evaluate cell subsets specificity. As a result, we found the specificity of Neu_ c1_ IL1B and Neu_ c2_ cxcr4 (low) in postoperative blood has increased, while that of IL-7R + neutrophils has decreased, indicating that these groups of cells possibly differentiated or migrated to other subgroups in the state of lung cancer. In addition, Neu_c3_CST7, RSAD2_Neu, S100A2/Pabpc1_Neu, ISG15/Ifit3_Neu, CD74_Neu, PTGS2/Actg1_Neu, SPP1_Neu were high specific in all the four NSCLC-associated samples. Meanwhile, we also integrated the single-cell sequencing results of other diseases from lung and various organs, in order to reach a more objective conclusion. IL-7R + neutrophils, Lgals1_mNeu, and CD74_Neu played an important role under pathological state. They generally existed in the tissues and circulating blood of patients with NSCLC, and their proportion in the tissues was particularly high. According to the OER value, the specificity of IL-7R + neurophils subgroup in circulating blood was higher, indicating that its physiological state changes and gene expression in blood may be more reliable. On the other hand, it also showed that the high proportion of cell subgroup expression did not mean accurate clustering.

As the main reason for the failure of targeted therapy and disease resistance, intra-tumor heterogenicity has always been a hot issue in the field of biology and medicine (Gay et al. 2016). ScRNA-seq is able to identify the changes of cell subpopulations under health and disease conditions based on the precise resolution ability. However, due to the complexity of biomarkers and the interconnection between cell groups, not all cell subgroups have specific gene panels for identification. Therefore, it is an important work to restore each cell subgroup as much as possible. We have developed a set of calculation methods for evaluating the distribution of cell subpopulations, and defined the specificity of subpopulations by OER values. This is necessary because the various gene panels used for cell grouping mentioned in the published literature may be non-specific. This non-specificity can lead to interference in the process of defining cell group types.

The evaluation system of specificity of subpopulations was developed to provide a novel tool for diagnosis and treatment. Firstly, subpopulation identification can be further standardized based on OER in single-cell analysis. In fact, we have been developing a set of methods through applying the specificity assessment system to identify cell subpopulations, in order to obtain a definitive UMAP. Briefly, the most specific subgroup would be the final choice if a certain cell is deemed adequate for two or more subpopulations based on the OER value. The feasibility and credibility of this approach are currently being evaluated. Secondly, changes of OER in different disease subtypes or status may suggest the differentiation or migration of a certain subset, which may be considered as potential biomarkers.

At the same time, we also acknowledge that there are still limitations of this method. The criterion for identifying subpopulations is that all markers in a certain gene panel are positive and the positivity proportion was above the threshold value. However, due to individual differences, the low expression of a certain marker may result in a missing of the whole gene panel. Whether to include these omitted subgroups needs to be fully considered, for lowering the threshold may result in a reduction in specificity. In addition, more samples of different physiological and pathological conditions need to be included to refine the existing results, and the analytical results should be validated through experiments in subsequent work.

In conclusion, comprehensive consideration of the distribution characteristics of neutrophil subsets helps to identify disease subtypes and assess the dynamic changes in immune activity.

## Supplementary Information

Below is the link to the electronic supplementary material.

Supplementary file1 (XLSX 84 KB)

## Data Availability

Data transparency: The datasets used or analyzed during the current study (except for that from public databases) are available from the corresponding author upon reasonable request.
